# An analysis of age-standardized suicide rates in Muslim-majority countries in 2000-2019

**DOI:** 10.1186/s12889-022-13101-3

**Published:** 2022-05-04

**Authors:** Bob Lew, David Lester, Kairi Kõlves, Paul S. F. Yip, Ying-Yeh Chen, Won Sun Chen, M. Tasdik Hasan, Harold G. Koenig, Zhi Zhong Wang, Muhamad Nur Fariduddin, Emek Yuce Zeyrek-Rios, Caryn Mei Hsien Chan, Feisul Mustapha, Mimi Fitriana, Housseini Dolo, Burak M. Gönültaş, Mahboubeh Dadfar, Mojtaba Davoudi, Ahmed M. Abdel-Khalek, Lai Fong Chan, Ching Sin Siau, Norhayati Ibrahim

**Affiliations:** 1 Australian Institute for Suicide Research and Prevention, School of Applied Psychology, Griffith University, Brisbane, Queensland Australia; 2 Stockton University, Galloway, New Jersey United States; 3 WHO Collaborating Centre for Research and Training in Suicide Prevention, Griffith University, Brisbane, Queensland Australia; 4 Hong Kong Jockey Club Center for Suicide Research and Prevention, University of Hong Kong, Hong Kong, China; 5 Taipei City Psychiatric Centre, Taipei City Hospital, Taipei, Taiwan; 6 School of Health Sciences, Swinburne University of Technology, Melbourne, Australia; 7 Jeeon Bangladesh Ltd., Dhaka, Bangladesh; 8 Department of Primary Care & Mental Health, University of Liverpool, Liverpool, United Kingdom; 9 Duke University Medical Center, Durham, NC USA; 10 King Abdulaziz University, Jeddah, Saudi Arabia; 11 Department of Epidemiology and Statistics, School of Public Health at Guangdong Medical University, Dongguan, Guangdong China; 12 Faculty of Education, Universiti Teknologi MARA, Bandar Puncak Alam, Selangor Darul Ehsan Malaysia; 13 Psychology Department, Mardin Artuklu University, Mardin, Turkey; 14 Centre for Community Health Studies (ReaCH), Faculty of Health Sciences, Universiti Kebangsaan Malaysia, Kuala Lumpur, Malaysia; 15 Non-Communicable Diseases Section, Disease Control Division, Ministry of Health, Putrajaya, Malaysia; 16 Department of Psychology, International University of Malaya-Wales, Kuala Lumpur, Malaysia; 17 Faculty of Nursing, Universitas Airlangga, Surabaya, Indonesia; 18 Filariasis Unit, Faculty of Medicine, Pharmacy and Dentistry, University of Bamako, Bamako, Mali; 19 Social Work Department., Faculty of Letters, Sivas Cumhuriyet University, Sivas, Turkey; 20 Department of Addiction, School of Behavioral Sciences and Mental Health (Tehran Institute of Psychiatry), Iran University of Medical Sciences, Tehran, Iran; 21 Social Determinants of Health Research Center, Mashhad University of Medical Sciences, Mashhad, Iran; 22 Department of Psychology, Faculty of Arts, Alexandria University, Alexandria, Egypt; 23 Department of Psychiatry, Faculty of Medicine, Universiti Kebangsaan Malaysia, Kuala Lumpur, Malaysia; 24 Centre for Healthy Ageing and Wellness (H-Care), Faculty of Health Sciences, Universiti Kebangsaan Malaysia, Kuala Lumpur, Malaysia; 25 Institute of Islam Hadhari, Universiti Kebangsaan Malaysia, Bangi, Malaysia

**Keywords:** Suicide rate, WHO Global Health Estimates, Islam, Joinpoint

## Abstract

**Background:**

This study examines the 20-year trend of suicide in 46 Muslim-majority countries throughout the world and compares their suicide rates and trends with the global average. Ecological-level associations between the proportion of the Muslim population, the age-standardized suicide rates, male-to-female suicide rate ratio, and the Human Development Index (HDI) in 2019 were examined.

**Methods:**

Age-standardized suicide rates were extracted from the WHO Global Health Estimates database for the period between 2000 and 2019. The rates in each country were compared with the age-standardized global average during the past 20 years. The countries were further grouped according to their regions/sub-regions to calculate the regional and sub-regional weighted age-standardized suicide rates involving Muslim-majority countries. Correlation analyses were conducted between the proportion of Muslims, age-standardized suicide rate, male: female suicide rate ratio, and the HDI in all countries. Joinpoint regression was used to analyze the age-standardized suicide rates in 2000-2019.

**Results:**

The 46 countries retained for analysis included an estimated 1.39 billion Muslims from a total worldwide Muslim population of 1.57 billion. Of these countries, eleven (23.9%) had an age-standardized suicide rate above the global average in 2019. In terms of regional/sub-regional suicide rates, Muslim-majority countries in the Sub-Saharan region recorded the highest weighted average age-standardized suicide rate of 10.02/100,000 population, and Southeastern Asia recorded the lowest rate (2.58/100,000 population). There were significant correlations between the Muslim population proportion and male-to-female rate ratios (r=-0.324, *p*=0.028), HDI index and age-standardized suicide rates (r=-0.506, *p*<0.001), and HDI index and male-to-female rate ratios (r=0.503, *p*<0.001) in 2019. Joinpoint analysis revealed that seven Muslim-majority countries (15.2%) recorded an increase in the average annual percentage change regarding age-standardized suicide rates during 2000-2019.

**Conclusions:**

Most Muslim-majority countries had lower age-standardized suicide rates than the global average, which might reflect religious belief and practice or due to Muslim laws in their judicial and social structure which may lead to underreporting. This finding needs further in-depth country and region-specific study with regard to its implication for public policy.

**Supplementary Information:**

The online version contains supplementary material available at 10.1186/s12889-022-13101-3.

## Background

In 2019, the World Health Organization (WHO) estimated over 700,000 suicide deaths globally, equalling an average age-standardized suicide rate of 9.0 per 100,000 population [1]. There are more than 20 suicide attempts per suicide [2]. Furthermore, it is estimated that 135 people are exposed to a suicide death [3]. The shared goal of the United Nations Sustainable Development Goals (SDGs) and the WHO’s Comprehensive Mental Health Action Plan 2013-2030 is to reduce the global suicide mortality rate by one-third by 2030 [1, 4, 5].

The global average suicide rate has been declining steadily for the past 20 years from 14.0 per 100,000 in 2000 to 9.0 in 2019 [1]. This decrease is partly explained by a notable decline in suicides in populous countries such as China and India, specifically from 14.9 in 2000 to 6.7 in 2019 (decline of 55.0%), and from 19.1 in 2000 to 12.9 in 2019 (decline of 32.5%), respectively. Furthermore, there was also a reduction of suicides in Europe. For example, a high suicide rate in Russia dropped by 55.8% from 48.9 per 100,000 in 2000 to 21.6 in 2019. However, suicide rates in the Americas increased. Nevertheless, a majority of suicide deaths (77%) still occur in Lower- and Middle-Income Countries (LMICs), including the majority of Muslim countries [1, 6, 7].

Religion has been identified as one of the protective factors of suicidality [8]. Lawrence et al. [9] argued that the protective effect of religion against suicidality depends on different dimensions of religion and may differ by type of suicidality (e.g., suicidal ideation vs. suicide attempt). A meta-analysis by Wu et al. [10] found that religion was protective against death by suicide, with greater protectiveness found in areas of higher religious homogeneity, western cultures, and older population. Lester’s review of the literature suggested that intrinsic religiosity was protective against suicidal ideation and attempt [11]. Dadfar et al. found that religiosity may have a positive impact on the suicidal behavior of Iranian psychiatric outpatients [12]. Another study among Malaysian adolescents found that those who reported higher spiritual well-being had lower levels of suicidal ideation [13].

There have been many studies indicating that suicide rates in Muslim (or Muslim-majority) countries are lower than in non-Muslim-majority countries. Lester [14] reviewed research in Muslim countries and found that the suicide rate was consistently lower in countries where Islam was the predominant religion, which has been further confirmed [15–18]. A recent study showed that Muslims have the lowest permissiveness level toward suicide among the major religions of the world independent of religiosity level, and permissiveness was correlated with national suicide rates [19]. In Shah and Chandia’s [20] cross-national study, countries with a lower proportion of Muslims were found to exhibit higher suicide rates in both males and females, which suggests the protective effect of Islam against suicide. However, Alothman and Fogarty [21] found that the median male-to-female suicide rate ratio among Muslim predominant countries was 2.5 in 2015, which was the second-highest after Christian predominant countries, at 3.3. It is unclear, however, whether there is a correlation between the proportion of Muslims and the male-to-female suicide rate ratio. There are exceptions to the low suicide rates recorded in Muslim-majority countries. Countries belonging to the former Soviet Union in Central Asia, despite being predominantly Muslim, have recorded high suicide rates, attributed to adverse economic, health service-related, and other societal factors [22].

In summary, previous research highlights that there may be a variety of factors associated with the suicide rates and male-to-female suicide rate ratios in Muslim majority countries. Nevertheless, there is a lack of studies on suicides in Muslim-majority countries, particularly on an aggregated level.

In 2020, the WHO made the estimates of age-standardized suicide rates from 2000 to 2019 available for its member states under the Global Health Estimates [1]. The WHO Global Health Estimates of suicide rates utilized information from the Global Burden of Disease study, the WHO Mortality Database, and other data sources to provide the best possible estimates. Bias from potential misclassification of suicide deaths was minimized by considering the potential contribution of undetermined and accidental deaths [23]. This information allows comparisons between Muslim-majority countries and with global and regional averages across two decades. Furthermore, we can further examine ecological-level associations between the age-standardized suicide rates, male-to-female suicide rate ratio, and other environmental factors. Therefore, the present study aimed to: (1) analyze the 20-year suicide trend in 46 Muslim majority countries and compare the trends with the global average; (2) compare the 2019 country suicide rates with the global average and their respective regions/sub-regions; (3) examine the association between the age-standardized suicide rates and the male-to-female suicide rate ratio, the proportion of Muslim population in a country, and the Human Development Index (HDI) in 2019. Based on previous research, we hypothesized that Muslim majority countries will have lower age-standardized suicide rates in comparison with the global and regional suicide rates. Further, we expected the proportion of Muslims in a country would be negatively correlated with the suicide rate.

## Methods

### Data source

The age-standardized suicide rates were extracted from the WHO Global Health Estimates database [1]. Age-standardized suicide rates were available for the period between 2000 and 2019. Population data were extracted from the United Nations Population Division in order to provide information regarding the population size of each country [24]. Index of each country on the Human Development Index (HDI) in 2019 was obtained from the United Nations Development Programme (UNDP) [25]. The HDI reflects the socioeconomic development of a country beyond just Gross Domestic Income, including also dimensions such as life expectancy, education index, and standard of living [25].

Muslim majority countries were identified if at least 50% of the population identified as Muslim [26]. The proportion of the Muslim population in a country was derived from World Population Review data [27]. A total of 49 countries were classified as Muslim majority countries based on the criterion above. Countries that were not simultaneously listed as UN member states and WHO member states were omitted. This resulted in the omission of three Muslim majority countries – Palestine, Western Sahara, and Mayotte (French protectorate).

The remaining 46 Muslim-majority countries were further grouped according to the United Nations geoscheme, a system dividing 249 countries and territories of the world into six regional and 22 sub-regional groups [28] (Table 1).

**Table 1:** List of 46 Muslim-majority countries, age-standardized suicide rate per 100,000 population, male-to-female suicide rate ratio, and Human Development Index ranking in 2019

Country	Estimated Muslim Population n (%)^a^	Suicide rate (both sexes) ^c^	Male to female suicide rate ratio^c^	Human Development Index (HDI)^d^	Regional age-standardized suicide rate (Muslim-majority countries only)	Regional age-standardized suicide rate (Other countries)	Regional age-standardized suicide rate (All countries)
Total	1392957160				-	-	-
**Central Asia**^b^					7.96	-	7.96
Uzbekistan	31827355 (96.5)	8.28	2.41	0.720			
Kazakhstan	13023102 (70.2)	18.05	4.48	0.825			
Tajikistan	9013429 (96.7)	5.32	2.15	0.668			
Turkmenistan	5543974 (93.3)	6.07	3.24	0.715			
Kyrgyzstan	5132681 (80.0)	8.28	3.86	0.697			
**South Asia**^b^					2.82	5.46	3.93
Pakistan	208985531 (96.5)	9.77	3.11	0.557			
Bangladesh	147393740 (90.4)	3.85	3.53	0.632			
Afghanistan	37889590 (99.6)	5.96	1.09	0.511			
Maldives	522462 (98.4)	2.76	4.56	0.740			
**Southeastern Asia**^b^					2.58	7.29	3.31
Indonesia	235985494 (87.2)	2.55	3.33	0.718			
Malaysia	19585221 (61.3)	5.77	3.75	0.810			
Brunei	341437 (78.8)	2.54	5.25	0.838			
**Western Asia**^b^					4.87	3.94	4.47
Iran	82416410 (99.4)	5.13	2.78	0.783			
Turkey	82762170 (99.2)	2.34	3.00	0.820			
Iraq	37619468 (95.7)	4.74	3.04	0.674			
Saudi Arabia	33274742 (97.1)	5.43	4.11	0.854			
Yemen	28899465 (99.1)	7.06	1.70	0.470			
Syria	15875223 (93.0)	2.11	4.38	0.567			
Jordan	9818849 (97.2)	1.98	3.33	0.729			
Azerbaijan	9736240 (96.9)	3.97	4.40	0.756			
UAE	7425600 (76.0)	5.24	2.42	0.890			
Lebanon	3955744 (57.7)	2.76	2.29	0.744			
Oman	4273518 (85.9)	4.47	5.82	0.813			
Kuwait	3138479 (74.6)	2.66	5.43	0.806			
Qatar	2194855 (77.5)	4.66	3.35	0.848			
Bahrain	1209538 (73.7)	7.20	4.30	0.852			
**North Africa**^b^					4.23	-	4.23
Egypt	92758582 (92.4)	3.41	2.09	0.707			
Algeria	42622523 (99.0)	2.60	1.74	0.748			
Sudan	41528840 (97.0)	4.76	1.91	0.510			
Morocco	36107048 (99.0)	7.29	2.15	0.686			
Tunisia	11671332 (99.8)	3.18	2.56	0.740			
Libya	6574129 (97.0)	4.49	2.10	0.724			
**Sub-Saharan Africa**^b^					10.02	10.79	10.68
Niger	22914437 (98.3)	10.15	2.20	0.394			
Mali	18675122 (95.0)	7.96	1.84	0.434			
Senegal	15660804 (96.1)	10.99	3.56	0.512			
Burkina Faso	12497651 (61.5)	14.38	3.77	0.452			
Somalia	15412020 (99.8)	14.66	3.21	-			
Guinea	11379180 (89.1)	12.33	2.30	0.477			
Chad	9249192 (58.0)	13.22	2.93	0.398			
Sierra Leone	6141181 (78.6)	11.25	1.80	0.452			
Mauritania	4525698 (100.0)	5.47	1.90	0.546			
Gambia	2246745 (95.7)	9.64	2.15	0.496			
Djibouti	944350 (97.0)	11.95	2.14	0.524			
Comoros	836426 (98.3)	8.46	1.95	0.554			
**Southern Europe**^b^					5.83	10.02	9.53
Bosnia & Herzegovina	1673606 (50.7)	8.25	3.97	0.780			
Albania	1693977 (58.8)	3.72	2.41	0.795			

*Note*. ^a^World Population Review. ^b^United Nations, SDG Indicators: Regional groupings used in report and Statistical Annex. ^c^WHO Global Health Observatory data. ^d^United Nations Development Programme

### Statistical analysis

In order to calculate regional average age-standardized suicide rates, the country-specific weight was calculated by using the country-specific population divided by the total population size for the region. Next, for each region, the weighted average age-standardized suicide rate was calculated as the sum of all suicide rates multiplied by their weights [29].

As the data were normally distributed (based on skewness and kurtosis values of < ±2 and < ±7 respectively [30]), Pearson’s correlation coefficients were calculated between the proportion of Muslims in a country, the suicide rate, male-to-female suicide rate ratio, and HDI in 2019.

Trend analyses of country suicide rates were conducted using Joinpoint regression, using Joinpoint version 4.8.0. Joinpoint regression was used to identify the occurrence of statistically significant changes in the trend, i.e. age-standardized suicide rate, where the slope of the linear function changes [31]. Joinpoint regression provides an estimate of the annual percentage change (APC) and the average annual percentage change (AAPC) to assess the annual change in the age-standardized suicide rates in the 46 Muslim-majority countries from 2000 to 2019, with 95% confidence intervals (95%CIs). A *p*-value of less than 0.05 was used to determine statistical significance for all two-tailed tests.

This study did not require approval by an ethics committee as published population-based data were used.

## Results

The 46 countries included for analysis have an estimated 1.39 billion Muslims from a total population of about 1.57 billion. The average proportion of Muslims making up the 46 countries was 89.4%, with a range of 50.7% to 100.0%. Seven UN regions/subregions were represented, with the greatest number of countries from Western Asia (14 countries) and least from Southern Europe (two countries) (Table 1).

**Fig. 1:**
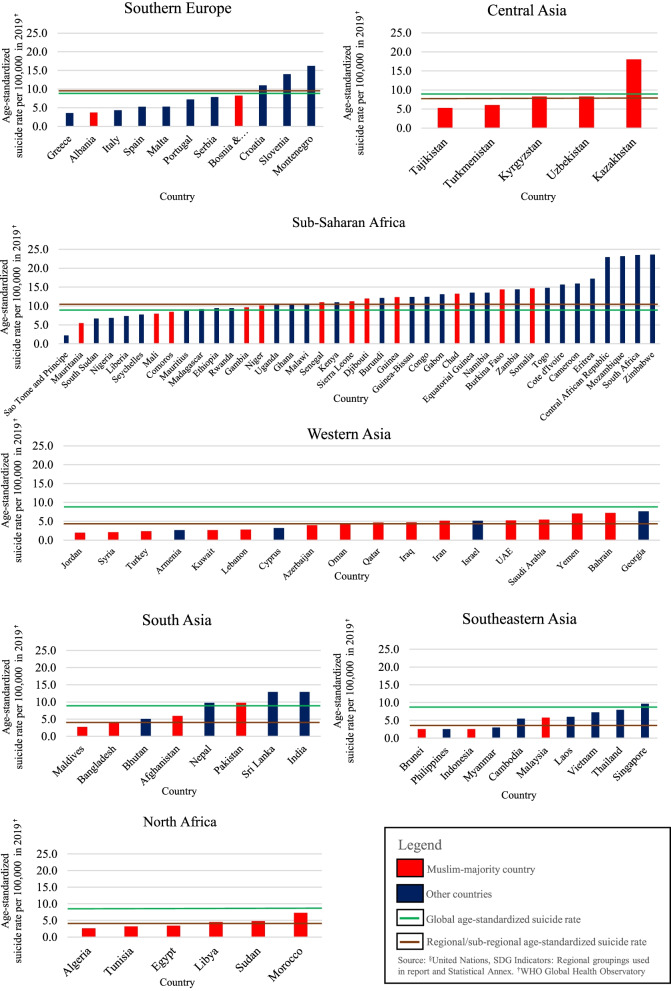
Age-standardized suicide rates of Muslim-majority and other countries based on region/sub-region in 2019. Source: WHO Global Health Observatory

Regarding the suicide rates of the Muslim-majority countries by region/sub-region, the Muslim-majority countries in the Sub-Saharan region recorded the highest weighted average age-standardized suicide rate of 10.02 per 100,000 population, followed by Central Asia (7.96), Southern Europe (5.83), Western Asia (4.87), North Africa (4.23), South Asia (2.82), and Southeastern Asia (2.58). In comparison with the global average, only Sub-Saharan Africa had an average rate that was higher than the global average. In terms of regional average, only Western Asia had an average rate that was higher than the regional average. In comparison with other (non-Muslim) countries in the region, only Western Asia recorded a higher average rate (Fig 1).

### The relationship between the proportion of Muslims, age-standardized suicide rate, male-to-female suicide rate ratio, and HDI in 2019

The results of the Pearson’s correlation showed that there was no relationship between the proportion of the Muslim population and the age-standardized suicide rate in Muslim-majority countries (*r*=-0.178 *p*=0.237). However, there was a significant correlation between the Muslim population proportion and male-to-female suicide rate ratio (*r*=-0.324, *p*=0.028). HDI index was significantly correlated with both, and age-standardized suicide rates (*r*=-0.506, *p*<0.001) and male-to-female suicide rate ratio (*r*=0.503, *p*<0.001).

### Suicide trends of Muslim-majority countries during the past 20 years

Globally, age-standardized suicide rates declined from 14.0 per 100,000 in year 2000 to 9.0 in year 2019, a decrease of 35.7%. Based on the Joinpoint analysis, the global trend had two Joinpoints in 2011 and 2015, where the trend showed a decline (AAPC: -2.3; 95%CI:-2.6, -2.0), with the decline becoming slower after 2015. Among the Muslim-majority countries analyzed, seven countries (Azerbaijan, Brunei, Guinea, Niger, Saudi Arabia, Sierra Leone, and Somalia; 15.2%) recorded a significant increase in AAPC.

### Muslim-majority countries in the Central Asia Sub-Region (6 countries)

Most of the Muslim-majority countries in Central Asia had a significant decrease in AAPC, except for Tajikistan which had a slight non-significant increase in AAPC of 0.3 (95%CI -0.1, 0.7). The largest decrease was recorded by Turkmenistan, with AAPC of –4.5 (95%CI -5.2, -3.9), and an overall decrease of 56.2%. Kazakhstan had one of the world’s highest suicide rates, and even though it recorded a decrease (AAPC: -3.8; 95%CI:-4.3 to -3.3), the rate of 18.1 in 2019 was still the highest among all the 46 Muslim-majority countries included for analysis.

### Muslim-majority countries in the South Asia Sub-Region (3 countries)

All Muslim-majority countries in the South Asia Sub-Region recorded a negative AAPC, and the suicide rates were below the global average over the past 20 years. Maldives had the greatest decline in suicide rate (47.9%) during the past 20 years (AAPC -3.4; 95% CI: -4.5, -2.4). Bangladesh had been recording a significant overall decrease in its suicide rate during the past 20 years (AAPC-3.0; 95%CI: -3.5, -2.6). However, an upward trend was detected from 2014 to 2019 (APC 1.6; 95%CI 0.7, 2.4).

### Muslim-majority countries in the Southeastern Asia Sub-Region (3 countries)

Muslim-majority countries in Southeastern Asia recorded the lowest suicide rates in comparison with the other six regions/sub-regions and all countries had rates below the world average. While Indonesia recorded a decline (AAPC -2.0; 95%CI: -2.2, -1.8), Brunei recorded an overall upward trend (AAPC 2.6; 95%CI:0.7, 4.5) from 2000 to 2019.

### Muslim-majority countries in the Western Asia Sub-Region (14 countries)

The suicide rate decreased significantly for all Muslim-majority countries in Western Asia, except for Azerbaijan (AAPC: 0.8; 95%CI:0.3 to 1.2) and Saudi Arabia (AAPC: 1.9; 95%CI:1.5 to 2.2). Saudi Arabia recorded the greatest increase in the past 20 years (41.4%) among all Western Asian Muslim-majority countries. All countries, however, trended below the global average rate during the past 20 years. Turkey recorded the largest decrease in suicide rate in the region (-44.4%). Kuwait and Syria recorded an upward trend for the years 2011-2019 and 2006-2019 respectively, even though Kuwait recorded an overall decrease of -14.2% during the past 20 years, whilst Syria recorded an overall increase of 5.0% during the same time period.

### Muslim-majority countries in the North Africa Sub-Region (6 countries)

All the Muslim-majority countries in the North Africa sub-region trended below the global average. Morocco had the highest suicide rates in this region during the 20 years of observation. Most countries recorded a decline in the AAPC, except for Libya, which recorded a non-significant AAPC of 0.1 (95%CI:-0.6, 0.9).

### Muslim-majority countries in the Sub-Saharan Africa Sub-Region (12 countries)

In 2019, the majority of Sub-Saharan African Muslim-majority countries recorded suicide rates above the global average, except Comoros (8.5), Mali (8.0), and Mauritania (5.5). Guinea and Niger both recorded an overall increase in AAPC (1.3; 95%CI: 1.0, 1.6 and 0.2; 95%CI: 0.0, 0.4 respectively), with an upward trend between 2000 to 2013, and a downward trend between 2013 to 2019. Similarly, Sierra Leone had an overall increase in suicide rates (AAPC 0.5; 95%CI: 0.0, 1.0), with multiple fluctuations.

### Muslim-majority countries in the Southern Europe Sub-Region (2 countries)

Albania showed a long-term decrease in the suicide rate from 5.2 in 2000 to 3.7 in 2019; a decrease of -28.9%. Bosnia and Herzegovina had a stable trend showing a small growth from 8.1 in 2000 to 8.3 in 2019, an increase of 1.5%. It fluctuated between 8.1 and 9.0 during this period, having two Joinpoints and showed an increasing trend in initial years until 2007 after which it declined for a few years before flattening from 2010 onward (AAPC: 0.0; 95%CI:-0.8 to 0.8) (Table 2; Fig S1 in Supplementary Material).

**Table 2:** Joinpoint analysis of the suicide trend of the global average and Muslim-majority countries from 2000 to 2019

**Global Average**							
Segment	Lower Endpoint	Upper Endpoint	APC	Lower CI	Upper CI	Test Statistic (t)	Prob > |t|
1	2000	2011	-2.2*	-2.4	-2.1	-29.0	< 0.001
2	2011	2015	-3.6*	-4.8	-2.4	-6.4	< 0.001
3	2015	2019	-0.9*	-1.7	-0.1	-2.6	0.024
Range	Lower Endpoint	Upper Endpoint	AAPC	Lower CI	Upper CI	Test Statistic~	*P*-Value~
Full Range	2000	2019	-2.3*	-2.6	-2.0	-15.1	< 0.05
**Central Asia** **Kazakhstan**							
Segment	Lower Endpoint	Upper Endpoint	APC	Lower CI	Upper CI	Test Statistic (t)	Prob > |t|
1	2000	2005	0.0	-1.8	1.9	0.0	0.990
2	2005	2019	-5.2*	-5.5	-4.8	-29.4	< 0.001
Range	Lower Endpoint	Upper Endpoint	AAPC	Lower CI	Upper CI	Test Statistic~	*P*-Value~
Full Range	2000	2019	-3.8*	-4.3	-3.3	-14.9	< 0.05
**Kyrgyzstan**							
Segment	Lower Endpoint	Upper Endpoint	APC	Lower CI	Upper CI	Test Statistic (t)	Prob > |t|
1	2000	2014	-2.9*	-3.2	-2.6	-18.7	< 0.001
2	2014	2019	-5.9*	-7.4	-4.3	-8.0	< 0.001
Range	Lower Endpoint	Upper Endpoint	AAPC	Lower CI	Upper CI	Test Statistic~	*P*-Value~
Full Range	2000	2019	-3.7*	-4.1	-3.3	-16.4	< 0.05
**Tajikistan**							
Segment	Lower Endpoint	Upper Endpoint	APC	Lower CI	Upper CI	Test Statistic (t)	Prob > |t|
1	2000	2002	-5.2*	-9.0	-1.2	-2.7	0.015
2	2002	2019	1.0*	0.8	1.1	14.4	< 0.001
Range	Lower Endpoint	Upper Endpoint	AAPC	Lower CI	Upper CI	Test Statistic~	*P*-Value~
Full Range	2000	2019	0.3	-0.1	0.7	1.5	>0.05
**Turkmenistan**							
Segment	Lower Endpoint	Upper Endpoint	APC	Lower CI	Upper CI	Test Statistic (t)	Prob > |t|
1	2000	2006	1.4*	0.2	2.6	2.6	0.024
2	2006	2011	-13.4*	-15.3	-11.5	-14.2	< 0.001
3	2011	2019	-3.0*	-3.7	-2.2	-8.6	< 0.001
Range	Lower Endpoint	Upper Endpoint	AAPC	Lower CI	Upper CI	Test Statistic~	*P*-Value~
Full Range	2000	2019	-4.5*	-5.2	-3.9	-13.2	< 0.05
**Uzbekistan**							
Segment	Lower Endpoint	Upper Endpoint	APC	Lower CI	Upper CI	Test Statistic (t)	Prob > |t|
1	2000	2009	-4.0*	-4.6	-3.4	-14.0	< 0.001
2	2009	2016	1.4*	0.3	2.6	2.7	0.020
3	2016	2019	-3.6*	-6.8	-0.2	-2.3	0.042
Range	Lower Endpoint	Upper Endpoint	AAPC	Lower CI	Upper CI	Test Statistic~	*P*-Value~
Full Range	2000	2019	-1.9*	-2.6	-1.3	-5.6	< 0.05
**South Asia** **Pakistan**							
Segment	Lower Endpoint	Upper Endpoint	APC	Lower CI	Upper CI	Test Statistic (t)	Prob > |t|
1	2000	2003	0.8	-0.2	1.7	1.8	0.101
2	2003	2011	-1.5*	-1.8	-1.3	-13.2	< 0.001
3	2011	2019	-0.2*	-0.4	-0.0	-2.4	0.034
Range	Lower Endpoint	Upper Endpoint	AAPC	Lower CI	Upper CI	Test Statistic~	*P*-Value~
Full Range	2000	2019	-0.6*	-0.8	-0.4	-6.6	< 0.05
**Bangladesh**							
Segment	Lower Endpoint	Upper Endpoint	APC	Lower CI	Upper CI	Test Statistic (t)	Prob > |t|
1	2000	2003	-5.9*	-7.6	-4.1	-7.2	< 0.001
2	2003	2008	-1.3*	-2.5	-0.1	-2.5	0.035
3	2008	2014	-6.7*	-7.5	-5.9	-18.5	< 0.001
4	2014	2019	1.6*	0.7	2.4	4.2	0.002
Range	Lower Endpoint	Upper Endpoint	AAPC	Lower CI	Upper CI	Test Statistic~	*P*-Value~
Full Range	2000	2019	-3.0*	-3.5	-2.6	-12.5	< 0.05
**Afghanistan**							
Segment	Lower Endpoint	Upper Endpoint	APC	Lower CI	Upper CI	Test Statistic (t)	Prob > |t|
1	2000	2006	-0.4	-1.1	0.3	-1.3	0.223
2	2006	2012	-3.5*	-4.4	-2.7	-8.8	< 0.001
3	2012	2019	-0.6*	-1.1	-0.1	-2.5	0.027
Range	Lower Endpoint	Upper Endpoint	AAPC	Lower CI	Upper CI	Test Statistic~	*P*-Value~
Full Range	2000	2019	-1.5*	-1.8	-1.1	-8.1	< 0.05
**Maldives**							
Segment	Lower Endpoint	Upper Endpoint	APC	Lower CI	Upper CI	Test Statistic (t)	Prob > |t|
1	2000	2004	-7.1*	-9.0	-5.2	-8.1	< 0.001
2	2004	2013	-4.1*	-4.8	-3.4	-13.4	< 0.001
3	2013	2016	4.9	-1.7	12.0	1.7	0.130
4	2016	2019	-4.3*	-7.4	-1.1	-3.0	0.014
Range	Lower Endpoint	Upper Endpoint	AAPC	Lower CI	Upper CI	Test Statistic~	*P*-Value~
Full Range	2000	2019	-3.4*	-4.5	-2.4	-6.2	< 0.05
**Southeastern Asia** **Indonesia**							
Segment	Lower Endpoint	Upper Endpoint	APC	Lower CI	Upper CI	Test Statistic (t)	Prob > |t|
1	2000	2014	-2.7*	-2.8	-2.5	-36.4	< 0.001
2	2014	2019	-0.3	-1.0	0.5	-0.8	0.439
Range	Lower Endpoint	Upper Endpoint	AAPC	Lower CI	Upper CI	Test Statistic~	*P*-Value~
Full Range	2000	2019	-2.0*	-2.2	-1.8	-19.1	< 0.05
**Malaysia**							
Segment	Lower Endpoint	Upper Endpoint	APC	Lower CI	Upper CI	Test Statistic (t)	Prob > |t|
1	2000	2013	-1.4*	-1.7	-1.1	-11.3	< 0.001
2	2013	2019	2.3*	1.5	3.2	5.7	< 0.001
Range	Lower Endpoint	Upper Endpoint	AAPC	Lower CI	Upper CI	Test Statistic~	*P*-Value~
Full Range	2000	2019	-0.2	-0.5	0.1	-1.5	>0.05
**Brunei**							
Segment	Lower Endpoint	Upper Endpoint	APC	Lower CI	Upper CI	Test Statistic (t)	Prob > |t|
1	2000	2019	2.6*	0.7	4.5	2.9	0.010
Range	Lower Endpoint	Upper Endpoint	AAPC	Lower CI	Upper CI	Test Statistic~	*P*-Value~
Full Range	2000	2019	2.6*	0.7	4.5	2.9	< 0.05
**Western Asia** **Iran**							
Segment	Lower Endpoint	Upper Endpoint	APC	Lower CI	Upper CI	Test Statistic (t)	Prob > |t|
1	2000	2006	-5.6*	-6.7	-4.5	-10.8	< 0.001
2	2006	2015	0.8*	0.1	1.6	2.4	0.036
3	2015	2019	-4.1*	-6.2	-2.0	-4.2	0.001
Range	Lower Endpoint	Upper Endpoint	AAPC	Lower CI	Upper CI	Test Statistic~	*P*-Value~
Full Range	2000	2019	-2.3*	-2.9	-1.7	-7.4	< 0.05
**Turkey**							
Segment	Lower Endpoint	Upper Endpoint	APC	Lower CI	Upper CI	Test Statistic (t)	Prob > |t|
1	2000	2007	-6.2*	-7.1	-5.2	-14.0	< 0.001
2	2007	2012	-2.8*	-5.0	-0.4	-2.6	0.024
3	2012	2019	0.3	-0.7	1.3	0.6	0.586
Range	Lower Endpoint	Upper Endpoint	AAPC	Lower CI	Upper CI	Test Statistic~	*P*-Value~
Full Range	2000	2019	-3.0*	-3.7	-2.2	-8.1	< 0.05
**Iraq**							
Segment	Lower Endpoint	Upper Endpoint	APC	Lower CI	Upper CI	Test Statistic (t)	Prob > |t|
1	2000	2008	-0.7*	-1.2	-0.3	-3.7	0.003
2	2008	2013	2.9*	1.6	4.2	4.9	< 0.001
3	2013	2019	-3.1*	-3.7	-2.4	-10.0	< 0.001
Range	Lower Endpoint	Upper Endpoint	AAPC	Lower CI	Upper CI	Test Statistic~	*P*-Value~
Full Range	2000	2019	-0.6*	-0.9	-0.2	-2.7	< 0.05
**Saudi Arabia**							
Segment	Lower Endpoint	Upper Endpoint	APC	Lower CI	Upper CI	Test Statistic (t)	Prob > |t|
1	2000	2005	1.0*	0.1	1.9	2.5	0.026
2	2005	2012	7.1*	6.4	7.8	23.3	< 0.001
3	2012	2019	-2.5*	-3.0	-2.0	-11.0	< 0.001
Range	Lower Endpoint	Upper Endpoint	AAPC	Lower CI	Upper CI	Test Statistic~	*P*-Value~
Full Range	2000	2019	1.9*	1.5	2.2	10.7	< 0.05
**Yemen**							
Segment	Lower Endpoint	Upper Endpoint	APC	Lower CI	Upper CI	Test Statistic (t)	Prob > |t|
1	2000	2015	-1.4*	-1.5	-1.3	-23.7	< 0.001
2	2015	2019	0.8	-0.2	1.7	1.7	0.113
Range	Lower Endpoint	Upper Endpoint	AAPC	Lower CI	Upper CI	Test Statistic~	*P*-Value~
Full Range	2000	2019	-1.0*	-1.2	-0.8	-9.1	< 0.05
**Syria**							
Segment	Lower Endpoint	Upper Endpoint	APC	Lower CI	Upper CI	Test Statistic (t)	Prob > |t|
1	2000	2006	-1.8	-3.8	0.2	-1.9	0.072
2	2006	2019	1.3*	0.7	1.9	4.4	< 0.001
Range	Lower Endpoint	Upper Endpoint	AAPC	Lower CI	Upper CI	Test Statistic~	*P*-Value~
Full Range	2000	2019	0.3	-0.4	1.0	0.9	>0.05
**Jordan**							
Segment	Lower Endpoint	Upper Endpoint	APC	Lower CI	Upper CI	Test Statistic (t)	Prob > |t|
1	2000	2004	-3.8*	-5.2	-2.4	-6.1	< 0.001
2	2004	2009	-7.2*	-8.5	-5.8	-11.8	< 0.001
3	2009	2015	-1.6*	-2.6	-0.6	-3.7	0.005
4	2015	2019	1.2	-0.3	2.6	1.9	0.096
Range	Lower Endpoint	Upper Endpoint	AAPC	Lower CI	Upper CI	Test Statistic~	*P*-Value~
Full Range	2000	2019	-3.0*	-3.5	-2.4	-10.5	< 0.05
**Azerbaijan**							
Segment	Lower Endpoint	Upper Endpoint	APC	Lower CI	Upper CI	Test Statistic (t)	Prob > |t|
1	2000	2006	6.5*	5.7	7.4	18.1	< 0.001
2	2006	2016	-2.5*	-2.9	-2.1	-13.3	< 0.001
3	2016	2019	0.7	-1.6	3.0	0.6	0.536
Range	Lower Endpoint	Upper Endpoint	AAPC	Lower CI	Upper CI	Test Statistic~	*P*-Value~
Full Range	2000	2019	0.8*	0.3	1.2	3.5	< 0.05
**United Arab Emirates**							
Segment	Lower Endpoint	Upper Endpoint	APC	Lower CI	Upper CI	Test Statistic (t)	Prob > |t|
1	2000	2003	-5.1*	-7.3	-2.9	-5.1	0.001
2	2003	2011	-1.0*	-1.6	-0.4	-3.6	0.005
3	2011	2017	-4.7*	-5.7	-3.7	-10.6	< 0.001
4	2017	2019	4.0	-0.7	9.0	1.9	0.085
Range	Lower Endpoint	Upper Endpoint	AAPC	Lower CI	Upper CI	Test Statistic~	*P*-Value~
Full Range	2000	2019	-2.3*	-3.0	-1.7	-7.2	< 0.05
**Lebanon**							
Segment	Lower Endpoint	Upper Endpoint	APC	Lower CI	Upper CI	Test Statistic (t)	Prob > |t|
1	2000	2019	-0.2	-0.6	0.1	-1.3	0.199
Range	Lower Endpoint	Upper Endpoint	AAPC	Lower CI	Upper CI	Test Statistic~	*P*-Value~
Full Range	2000	2019	-0.2	-0.6	0.1	-1.3	>0.05
**Oman**							
Segment	Lower Endpoint	Upper Endpoint	APC	Lower CI	Upper CI	Test Statistic (t)	Prob > |t|
1	2000	2010	-0.8*	-1.5	-0.2	-2.8	0.016
2	2010	2013	-8.2*	-15.6	-0.0	-2.2	0.049
3	2013	2019	-1.0	-2.4	0.5	-1.5	0.168
Range	Lower Endpoint	Upper Endpoint	AAPC	Lower CI	Upper CI	Test Statistic~	*P*-Value~
Full Range	2000	2019	-2.1*	-3.3	-0.8	-3.1	< 0.05
**Kuwait**							
Segment	Lower Endpoint	Upper Endpoint	APC	Lower CI	Upper CI	Test Statistic (t)	Prob > |t|
1	2000	2005	-2.4*	-4.6	-0.2	-2.5	0.036
2	2005	2008	2.7	-7.1	13.5	0.6	0.561
3	2008	2011	-7.9	-16.7	1.8	-1.9	0.096
4	2011	2019	2.3*	1.2	3.4	4.7	0.001
Range	Lower Endpoint	Upper Endpoint	AAPC	Lower CI	Upper CI	Test Statistic~	*P*-Value~
Full Range	2000	2019	-0.6	-2.6	1.5	-0.5	1
**Qatar**							
Segment	Lower Endpoint	Upper Endpoint	APC	Lower CI	Upper CI	Test Statistic (t)	Prob > |t|
1	2000	2005	-4.4*	-5.8	-2.9	-6.5	< 0.001
2	2005	2008	4.1	-2.9	11.5	1.3	0.222
3	2008	2015	-5.3*	-6.4	-4.2	-10.6	< 0.001
4	2015	2019	-1.2	-3.3	1.0	-1.3	0.241
Range	Lower Endpoint	Upper Endpoint	AAPC	Lower CI	Upper CI	Test Statistic~	*P*-Value~
Full Range	2000	2019	-2.8*	-3.9	-1.6	-4.8	< 0.05
**Bahrain**							
Segment	Lower Endpoint	Upper Endpoint	APC	Lower CI	Upper CI	Test Statistic (t)	Prob > |t|
1	2000	2003	11.5*	3.5	20.2	3.2	0.008
2	2003	2013	-4.9*	-6.2	-3.6	-8.0	< 0.001
3	2013	2019	2.3	-0.2	4.9	2.0	0.071
Range	Lower Endpoint	Upper Endpoint	AAPC	Lower CI	Upper CI	Test Statistic~	*P*-Value~
Full Range	2000	2019	-0.2	-1.6	1.2	-0.3	1
**North Africa** **Egypt**							
Segment	Lower Endpoint	Upper Endpoint	APC	Lower CI	Upper CI	Test Statistic (t)	Prob > |t|
1	2000	2008	-0.7*	-1.4	-0.1	-2.5	0.027
2	2008	2013	1.6	-0.3	3.5	1.8	0.090
3	2013	2019	-2.2*	-3.1	-1.2	-4.8	< 0.001
Range	Lower Endpoint	Upper Endpoint	AAPC	Lower CI	Upper CI	Test Statistic~	*P*-Value~
Full Range	2000	2019	-0.6*	-1.1	-0.0	-2.0	< 0.05
**Algeria**							
Segment	Lower Endpoint	Upper Endpoint	APC	Lower CI	Upper CI	Test Statistic (t)	Prob > |t|
1	2000	2011	-4.4*	-4.6	-4.1	-39.9	< 0.001
2	2011	2014	-1.0	-4.7	2.8	-0.6	0.552
3	2014	2017	-3.9*	-7.4	-0.2	-2.4	0.041
4	2017	2019	2.2	-1.6	6.1	1.3	0.225
Range	Lower Endpoint	Upper Endpoint	AAPC	Lower CI	Upper CI	Test Statistic~	*P*-Value~
Full Range	2000	2019	-3.1*	-3.9	-2.3	-7.6	< 0.05
**Sudan**							
Segment	Lower Endpoint	Upper Endpoint	APC	Lower CI	Upper CI	Test Statistic (t)	Prob > |t|
1	2000	2008	-0.8*	-1.2	-0.4	-5.0	0.001
2	2008	2011	0.4	-3.0	3.9	0.3	0.795
3	2011	2016	-1.7*	-2.8	-0.7	-3.6	0.005
4	2016	2019	-0.1	-1.8	1.7	-0.1	0.913
Range	Lower Endpoint	Upper Endpoint	AAPC	Lower CI	Upper CI	Test Statistic~	*P*-Value~
Full Range	2000	2019	-0.8*	-1.4	-0.2	-2.5	< 0.05
**Morocco**							
Segment	Lower Endpoint	Upper Endpoint	APC	Lower CI	Upper CI	Test Statistic (t)	Prob > |t|
1	2000	2011	-1.2*	-1.4	-0.9	-10.2	< 0.001
2	2011	2015	-5.6*	-7.4	-3.9	-6.7	< 0.001
3	2015	2019	-0.8	-2.0	0.4	-1.4	0.174
Range	Lower Endpoint	Upper Endpoint	AAPC	Lower CI	Upper CI	Test Statistic~	*P*-Value~
Full Range	2000	2019	-2.1*	-2.5	-1.6	-9.2	< 0.05
**Tunisia**							
Segment	Lower Endpoint	Upper Endpoint	APC	Lower CI	Upper CI	Test Statistic (t)	Prob > |t|
1	2000	2003	-0.1	-1.3	1.2	-0.1	0.929
2	2003	2016	-2.1*	-2.2	-1.9	-30.3	< 0.001
3	2016	2019	2.3*	1.0	3.6	4.0	0.002
Range	Lower Endpoint	Upper Endpoint	AAPC	Lower CI	Upper CI	Test Statistic~	*P*-Value~
Full Range	2000	2019	-1.1*	-1.3	-0.8	-7.7	< 0.05
**Libya**							
Segment	Lower Endpoint	Upper Endpoint	APC	Lower CI	Upper CI	Test Statistic (t)	Prob > |t|
1	2000	2019	0.1	-0.6	0.9	0.4	0.670
Range	Lower Endpoint	Upper Endpoint	AAPC	Lower CI	Upper CI	Test Statistic~	*P*-Value~
Full Range	2000	2019	0.1	-0.6	0.9	0.4	1
**Sub-Saharan Africa** **Niger**							
Segment	Lower Endpoint	Upper Endpoint	APC	Lower CI	Upper CI	Test Statistic (t)	Prob > |t|
1	2000	2013	0.8*	0.6	1.0	8.9	< 0.001
2	2013	2019	-0.9*	-1.5	-0.3	-3.3	0.005
Range	Lower Endpoint	Upper Endpoint	AAPC	Lower CI	Upper CI	Test Statistic~	*P*-Value~
Full Range	2000	2019	0.2*	0.0	0.4	2.2	< 0.05
**Mali**							
Segment	Lower Endpoint	Upper Endpoint	APC	Lower CI	Upper CI	Test Statistic (t)	Prob > |t|
1	2000	2013	0.1	-0.1	0.3	0.8	0.453
2	2013	2019	-1.8*	-2.4	-1.2	-6.3	< 0.001
Range	Lower Endpoint	Upper Endpoint	AAPC	Lower CI	Upper CI	Test Statistic~	*P*-Value~
Full Range	2000	2019	-0.5*	-0.7	-0.3	-4.8	< 0.05
**Senegal**							
Segment	Lower Endpoint	Upper Endpoint	APC	Lower CI	Upper CI	Test Statistic (t)	Prob > |t|
1	2000	2019	-1.3*	-1.4	-1.2	-27.2	< 0.001
Range	Lower Endpoint	Upper Endpoint	AAPC	Lower CI	Upper CI	Test Statistic~	*P*-Value~
Full Range	2000	2019	-1.3*	-1.4	-1.2	-27.2	< 0.05
**Burkina Faso**							
Segment	Lower Endpoint	Upper Endpoint	APC	Lower CI	Upper CI	Test Statistic (t)	Prob > |t|
1	2000	2002	-3.0*	-4.4	-1.7	-5.0	0.001
2	2002	2010	-0.1	-0.3	0.1	-1.4	0.195
3	2010	2013	0.6	-0.8	2.1	1.0	0.330
4	2013	2019	-1.8*	-2.0	-1.6	-17.3	< 0.001
Range	Lower Endpoint	Upper Endpoint	AAPC	Lower CI	Upper CI	Test Statistic~	*P*-*P*-Value~
Full Range	2000	2019	-0.8*	-1.1	-0.6	-6.6	< 0.05
**Somalia**							
Segment	Lower Endpoint	Upper Endpoint	APC	Lower CI	Upper CI	Test Statistic (t)	Prob > |t|
1	2000	2010	1.8*	1.4	2.2	10.4	< 0.001
2	2010	2015	-2.8*	-4.2	-1.3	-4.1	0.002
3	2015	2019	1.5*	0.0	3.1	2.2	0.047
Range	Lower Endpoint	Upper Endpoint	AAPC	Lower CI	Upper CI	Test Statistic~	*P*-Value~
Full Range	2000	2019	0.5*	0.0	1.0	2.1	< 0.05
**Guinea**							
Segment	Lower Endpoint	Upper Endpoint	APC	Lower CI	Upper CI	Test Statistic (t)	Prob > |t|
1	2000	2013	2.6*	2.3	2.9	21.3	< 0.001
2	2013	2019	-1.4*	-2.3	-0.6	-3.8	0.002
Range	Lower Endpoint	Upper Endpoint	AAPC	Lower CI	Upper CI	Test Statistic~	*P*-Value~
Full Range	2000	2019	1.3*	1.0	1.6	8.8	< 0.05
**Chad**							
Segment	Lower Endpoint	Upper Endpoint	APC	Lower CI	Upper CI	Test Statistic (t)	Prob > |t|
1	2000	2012	0.0	-0.2	0.2	0.1	0.937
2	2012	2015	-3.9*	-6.7	-1.0	-2.9	0.013
3	2015	2019	-1.2*	-2.2	-0.3	-2.9	0.014
Range	Lower Endpoint	Upper Endpoint	AAPC	Lower CI	Upper CI	Test Statistic~	*P*-Value~
Full Range	2000	2019	-0.9*	-1.3	-0.4	-3.7	< 0.05
**Sierra Leone**							
Segment	Lower Endpoint	Upper Endpoint	APC	Lower CI	Upper CI	Test Statistic (t)	Prob > |t|
1	2000	2010	1.8*	1.4	2.2	10.4	< 0.001
2	2010	2015	-2.8*	-4.2	-1.3	-4.1	0.002
3	2015	2019	1.5*	0.0	3.1	2.2	0.047
Range	Lower Endpoint	Upper Endpoint	AAPC	Lower CI	Upper CI	Test Statistic~	*P*-Value~
Full Range	2000	2019	0.5*	0.0	1.0	2.1	< 0.05
**Mauritania**							
Segment	Lower Endpoint	Upper Endpoint	APC	Lower CI	Upper CI	Test Statistic (t)	Prob > |t|
1	2000	2011	-1.4*	-1.7	-1.2	-10.8	< 0.001
2	2011	2019	0.1	-0.3	0.6	0.6	0.548
Range	Lower Endpoint	Upper Endpoint	AAPC	Lower CI	Upper CI	Test Statistic~	*P*-Value~
Full Range	2000	2019	-0.8*	-1.0	-0.5	-6.5	< 0.05
**Gambia**							
Segment	Lower Endpoint	Upper Endpoint	APC	Lower CI	Upper CI	Test Statistic (t)	Prob > |t|
1	2000	2003	1.4	-0.7	3.5	1.5	0.169
2	2003	2013	-0.8*	-1.2	-0.5	-4.9	< 0.001
3	2013	2019	-1.9*	-2.6	-1.3	-6.2	< 0.001
Range	Lower Endpoint	Upper Endpoint	AAPC	Lower CI	Upper CI	Test Statistic~	*P*-Value~
Full Range	2000	2019	-0.8*	-1.2	-0.5	-4.2	< 0.05
**Djibouti**							
Segment	Lower Endpoint	Upper Endpoint	APC	Lower CI	Upper CI	Test Statistic (t)	Prob > |t|
1	2000	2004	0.4	-0.9	1.7	0.7	0.512
2	2004	2008	-1.5	-3.6	0.5	-1.7	0.124
3	2008	2013	1.2	-0.1	2.6	2.1	0.063
4	2013	2019	0.0	-0.6	0.7	0.2	0.879
Range	Lower Endpoint	Upper Endpoint	AAPC	Lower CI	Upper CI	Test Statistic~	*P*-Value~
Full Range	2000	2019	0.1	-0.5	0.7	0.3	1
**Comoros**							
Segment	Lower Endpoint	Upper Endpoint	APC	Lower CI	Upper CI	Test Statistic (t)	Prob > |t|
1	2000	2005	-3.9*	-5.1	-2.7	-6.9	< 0.001
2	2005	2019	-0.4*	-0.7	-0.2	-3.4	0.004
Range	Lower Endpoint	Upper Endpoint	AAPC	Lower CI	Upper CI	Test Statistic~	*P*-Value~
Full Range	2000	2019	-1.3*	-1.7	-1.0	-7.7	< 0.05
**Southern Europe** **Bosnia and Herzegovina**							
Segment	Lower Endpoint	Upper Endpoint	APC	Lower CI	Upper CI	Test Statistic (t)	Prob > |t|
1	2000	2007	1.6*	0.9	2.3	5.2	< 0.001
2	2007	2010	-3.3	-8.1	1.7	-1.4	0.174
3	2010	2019	-0.1	-0.5	0.4	-0.4	0.711
Range	Lower Endpoint	Upper Endpoint	AAPC	Lower CI	Upper CI	Test Statistic~	*P*-Value~
Full Range	2000	2019	0.0	-0.8	0.8	0.1	1
**Albania**							
Segment	Lower Endpoint	Upper Endpoint	APC	Lower CI	Upper CI	Test Statistic (t)	Prob > |t|
1	2000	2008	8.1*	3.4	12.9	3.7	0.002
2	2008	2019	-7.9*	-10.4	-5.3	-6.4	< 0.001
Range	Lower Endpoint	Upper Endpoint	AAPC	Lower CI	Upper CI	Test Statistic~	*P*-Value~
Full Range	2000	2019	-1.5	-3.7	0.8	-1.3	>0.05

*Note*. *APC* Average Percent of Change. *AAPC* Annual Average Percentage of Change. *CI* Confidence Intervals. *UAE* United Arab Emirates. *significant at *p* < 0.05

## Discussion

The release of the new WHO estimates provided an opportunity to examine suicide rates across the globe including countries with relatively limited information. Therefore, the aim of this paper was to analyze the age-standardized suicide rates in 46 Muslim-majority countries from 2000 to 2019, and to compare the suicide trends with the global average. In addition, we compared the 2019 suicide rates with the global average and their respective regions/sub-regions; and examined the association between the age-standardized suicide rates and the male-to-female suicide rate ratio, with the proportion of Muslim population in a country, and the Human Development Index (HDI) in 2019. Our study found that the majority of the Muslim-majority countries (76.1%) had their average suicide rates below the global average in 2019. Despite some fluctuations, most of the countries (63.0%) showed an overall decline during the past 20 years (2000-2019).

### Suicide rates in Muslim-majority countries across regions and sub-regions

Of the 46 Muslim-majority countries analyzed, 11 (23.9%) had an age-standardized suicide rate above the global average in 2019. Of the 11 countries, nine are located in Sub-Saharan Africa, including Burkina Faso, Chad, Comoros, Djibouti, Gambia, Guinea, Mali, Mauritania, Niger, Senegal, Sierra Leone, and Somalia. The other two are in South Asia and Central Asia, i.e., Pakistan and Kazakhstan. These 11 countries are estimated to have about 318.5 million Muslims out of the total Muslim population of about 1,393.0 million (22.9%). The remaining 35 Muslim-majority countries have age-standardized suicide rates below the global average, and they consist of about 1,074.5 million Muslim populations comprising 77.1% of total Muslim populations located in the Muslim majority countries included in the analysis.

Traditionally, Kazakhstan has had high suicide rates and showed a twice higher rate compared to the global average in 2019. A recent study found that unemployment was the factor most strongly correlated with suicide rates in Kazakhstan between 2000 and 2019 [32]. The suicide rate estimated in Pakistan was also slightly above the global average. A scoping review on suicidal behavior and self-harm in Pakistan found that individuals below 30 years old and males to be at higher risk of suicide in Pakistan [33]. There is a lack of suicide research from Africa to explain the high suicide rates of nine Muslim majority countries in Africa, however, the WHO estimates show that the African region has the highest average suicide rate in 2019 [34].

Based on the country-level comparisons, the age-standardized suicide rates of most Muslim-majority countries were consistently lower than the global average during the past 20 years. This could be partly explained by the role of their religion and may also be due to some countries integrating Islamic principles into their governance and social systems. Suicide is prohibited under Islamic law, based on evidence from the Qur’an, the Sunnah, and the consensus of Muslim scholars. The person who dies by suicide is liable to be eternally condemned and experience God’s wrath. According to the Qur’an, in the 4^th^ surah, verse 29, “You shall not kill yourselves. Surely Allah is ever compassionate to you.” Apart from condemning suicide, the Qur’an emphasizes the sanctity of life, providing instructive guidance on the value of life and fulfilling the role as a human in this world whilst maintaining steadfastness, patience, and stability in all aspects of life (Qur’an, 17:33) [35]. In addition, adherence to the normative structures of collectivism such as cohesive communities, familial support, and collective goals together with religious commitment is important, and these may be protective against suicide [20]. Based on the above, the low suicide rates in most of the Muslim-majority countries may be related to the understanding of the Islamic concept of life within the matrix of Islamic thought such as legal thought, the Qur’an, and Prophetic Traditions. The findings are consistent with other studies reporting the protective effects of Islam in terms of reducing country- or region-wide suicide rates in Muslim countries [14–18].

On the other hand, suicide is also a major stigma in Muslim countries, which may lead to a reluctance to report a suicide death, and this may lead to the lower suicide rates in these countries [6, 36, 37]. The low rates of suicide in Muslim-majority countries which can be seen as a result of Islamic thought condemning taking one’s own life is not necessarily indicative of lower levels of suicidal ideation [38]. The suicidal ideation can manifest itself as non-fatal suicidal behavior or might be repressed by one’s religious belief. This is supported by higher attempted suicide rates in Ankara, Turkey [39], a high percentage of Turkish students considering suicide [40], and higher scores obtained in the suicide probability scale by Turkish university students compared to their American counterparts [38].

There were considerable cross-regional differences in the average age-standardized suicide rates in the seven regions and sub-regions of Central Asia, South Asia, Southeastern Asia, Western Asia, North Africa, Sub-Saharan Africa, and Southern Europe examined here. As a region, Sub-Saharan Africa recorded the highest average suicide rate among all the regions examined. According to Lester et al., gender inequalities may play a role in higher suicide rates in Africa and family power dynamics and domestic violence may contribute to this [41]. A narrative synthesis by Vijayakumar et al. further suggested that the suicide rate among African refugees was higher [42]. As Africa is home to the largest number of refugees, suicide among refugees might be an important factor behind the increasing overall rate of suicide deaths in African countries. In Sub-Saharan Africa, a meta-analysis among young people revealed that the median lifetime prevalence estimate of self-harm was 10.3% [43]. The high prevalence of self-harm may translate into a high suicide rate in this region.

In Western Asia, Muslim-majority countries recorded a higher average suicide rate than the overall Western Asia regional average, which included Muslim-majority and other countries. Of note, six out of 14 countries in this region recorded a male-to-female suicide rate ratio of more than 4.0. Future studies could expand on these preliminary findings to examine the reason behind the relatively higher suicide rates in Muslim-majority countries in the Western Asia region in comparison with other countries.

We found no association between the proportion of Muslims and age-standardized suicide rates. This finding is not consistent with Shah and Chandia’s [20] study on suicide rates of 27 countries based on WHO data between 1991-2002, where the higher proportion of Muslims was associated with lower suicide rates for both males and females. The difference may be because Shah and Chandia’s [20] study included countries where Muslims were not the majority. Therefore, further studies are needed to establish the protective effect of Islam for males and females. However, we found that the higher the proportion of Muslims in a country, the lower the male-to-female suicide rate ratio. This means females had a relatively higher risk of dying by suicide in Muslim-majority countries with a higher Muslim population proportion.

We also found that a higher HDI index was associated with a higher male-to-female suicide rate ratio, i.e. females had a lower risk of dying by suicide in countries with better quality of life and economic development. The findings were consistent with another study of 91 countries where a higher HDI was associated with higher suicide rates among males [44]. Factors contributing to higher female suicide rates such as greater gender inequality and lack of education and economic freedom [45] may be lower in Muslim-majority countries with a higher HDI. Our study also found that the higher the HDI index, the lower the age-standardized suicide rate in Muslim-majority countries. The results were dissimilar with another study in which high and very high HDI countries had significantly higher suicide rates in comparison with medium HDI countries [44]. However, the aforesaid study [44] did not include low HDI countries, which are more prevalent in our study. Therefore, HDI may be more protective of suicide among low and middle HDI countries in comparison with high and very high HDI countries, but this needs to be further investigated in future studies.

### Suicide trends in Muslim-majority countries across regions

Overall, the suicide trend in a majority of Muslim-majority countries indicated a significant decrease in 2000-2019. This was shown for countries in Central Asia which have traditionally recorded very high suicide rates and, nevertheless, even after a significant decrease, they still recorded higher rates than the global average. However, a few countries in Western Asia (Azerbaijan and Saudi Arabia) and Brunei in Southeastern Asia recorded an overall increase in the AAPCs during the past 20 years. In addition, a few countries experienced an upward trend after an initial decrease, which were Bangladesh (2014 onwards), Kuwait (2011 onwards), and Syria (2006 onwards). Syria recorded an upward trend between 2000-2010, a downward trend between 2010-2015, and an upward trend between 2015-2019. While it is difficult to determine the causes of these trends, a number of legal, health, and psychosocial events may be contributing factors. In Bangladesh, the ban of class I pesticides in 2000 was successful in decreasing suicide deaths attributable to pesticide poisoning between 2001 and 2014 [46]. However, a psychological autopsy study conducted on suicide deaths between July 2019 to July 2020 in Dhaka, Bangladesh showed that pesticide poisoning was still the most prevalent suicide method [47]. The upward trend in suicide rates in Syria between 2006 to 2019 may be partly explained by the Syrian civil war which began in 2011 and was a factor contributing to between 16% to 84% of Syrians suffering from post-traumatic stress disorder, and 11% to 49% from depression [48]. Sierra Leone recorded an upward trend in suicide rates between 2015 and 2019, and this may be related to the increased incidence of grief, post-traumatic stress, depression, and unexplained somatic symptoms owing to the Ebola outbreak between 2015 and 2017 [49]. These may have stemmed from the social ramifications of the disease such as the loss of loved ones, and stigma and violence against those presumed infected, depriving affected individuals of the traditional social support needed to overcome the crisis [50, 51].

The results of this study have implications for suicide prevention in Muslim-majority countries. First of all, based on the WHOMiNDBank, only Uzbekistan has a national suicide prevention policy [52]. In Kazakhstan, the Adolescent Mental Health and Suicide Prevention (AMHSP) program was implemented in two regions and incorporated within the 2015-2020 National Action Plan. The multisectoral collaboration with UNICEF Kazakhstan and a strong national agenda on suicide prevention resulted in falling suicide rates among adolescents aged 15-19 years old and is now being scaled nationally [53, 54]. There is a need for similar national suicide prevention activities to be developed and implemented in the other Muslim-majority countries. In addition, the considerable differences in suicide rates between the Muslim-majority countries studied should encourage further research on the multifaceted influence (or limitations) of religion in affecting the country- and region-level suicide rates [55]. For example, the countries represented in this study are from different socio-economic stages of development. How religious influence interacts with socio-economic and other factors is still far from definitive. Hence, any endeavor which attempts to provide a common explanation is bound to suffer from shortcomings and criticism. Future research can benefit from investigating the underlying mechanisms behind the decrease of suicide rates in countries that recorded a significant decrease in suicide rates during the past 20 years.

### Limitations

This was an analysis based on WHO estimated suicide data for each country. Of the 46 Muslim-majority countries surveyed, only three countries (Kazakhstan, Kuwait, and Kyrgyzstan) had vital registration data which was ranked high-quality, whilst 56.5% of the Muslim-majority countries, and all Muslim-majority countries from the Sub-Saharan Africa region, had death registration data which were either unavailable or deemed unusable due to their low quality. Therefore, these data need to be interpreted with caution [1]. Certain countries not listed as UN and WHO member states, such as Palestine, were not included in this study, as data on suicide rates were not available. Important insights from these countries may therefore be left out, and future studies are required. There was a wide range in the proportion of the population in each country that had a Muslim affiliation. No research has been done on the level of belief-specific practices followed in each country, which was beyond the scope of this paper. In addition, we did not disaggregate the 20-year suicide trend data by gender, age cohort, and other demographic indicators. This is recommended for future studies, which could contribute to a greater understanding of groups that contributed most to the changes, and therefore prevention initiatives could be tailored. The correlation analyses conducted between the proportion of the Muslim population, age-standardized suicide rate, male-to-female suicide rate ratio, and HDI did not control for other variables and was limited to 2019. Future studies should conduct panel analyses using multiple sources spanning several years.

The influence of Islamic culture or Islamic laws on gender roles was also not examined. Some countries may be governed by Syariah (Islamic) laws that may prohibit attempted suicide, including some which may criminalize attempted suicide. Effects of this on suicide rates were not investigated. Some countries may have under-reported suicide due to different levels of development and accuracy in their suicide reporting system. It is a custom for a Muslim to be buried early before decay sets in [56], normally, before the next day prayer. This practice may create pressure on the proper recording of suicide data as sometimes it limits the time available for the police, coroners, or pathologists to confirm the cause of death. Sudden death among women was also found to be less frequently reported as a suicide, in comparison with male suicide, due to the need to preserve family honor [57]. The WHO classified the mortality data quality of some countries as “poor”, and these need to be interpreted with caution [58].

## Conclusions

In conclusion, not all Muslim-majority countries have a lower suicide rate than the global average. This might be a reflection of the variations in the religious practice or the implementation of Syariah laws in judicial and social structures. This important finding needs further in-depth country- or region-specific exploration with policy analysis.

## Supplementary Information

**Additional file 1: Fig S1**. Age-standardized suicide rate in Muslim-majority countries in 2000-2019 and comparison with the global average.

## Data Availability

The datasets generated and/or analyzed during the current study are available in the WHO Global Health Estimates repository, https://www.who.int/data/gho/data/indicators/indicator-details/GHO/age-standardized-suicide-rates-(per-100-000-population)

## Abbreviations

AAPC: Average annual percentage change
APC: Annual percentage change
GHO: Global Health Observatory
HDI: Human Development Index
UNICEF: United Nations Children’s Fund
WHO: World Health Organization
