# Correction: An analysis of age-standardized suicide rates in Muslim-majority countries in 2000-2019

**DOI:** 10.1186/s12889-022-14136-2

**Published:** 2022-09-20

**Authors:** Bob Lew, David Lester, Kairi Kõlves, Paul S. F. Yip, Ying-Yeh Chen, Won Sun Chen, M. Tasdik Hasan, Harold G. Koenig, Zhi Zhong Wang, Muhamad Nur Fariduddin, Emek Yuce Zeyrek-Rios, Caryn Mei Hsien Chan, Feisul Mustapha, Mimi Fitriana, Housseini Dolo, Burak M. Gönültaş, Mahboubeh Dadfar, Mojtaba Davoudi, Ahmed M. Abdel-Khalek, Lai Fong Chan, Ching Sin Siau, Norhayati Ibrahim

**Affiliations:** 1 Australian Institute for Suicide Research and Prevention, School of Applied Psychology, Griffith University, Brisbane, Queensland Australia; 2 Stockton University, Galloway, New Jersey United States; 3 WHO Collaborating Centre for Research and Training in Suicide Prevention, Griffith University, Brisbane, Queensland Australia; 4 Hong Kong Jockey Club Center for Suicide Research and Prevention, University of Hong Kong, Hong Kong, China; 5 Taipei City Psychiatric Centre, Taipei City Hospital, Taipei, Taiwan; 6 School of Health Sciences, Swinburne University of Technology, Melbourne, Australia; 7 Jeeon Bangladesh Ltd., Dhaka, Bangladesh; 8 Department of Primary Care & Mental Health, University of Liverpool, Liverpool, United Kingdom; 9 Duke University Medical Center, Durham, NC USA; 10 King Abdulaziz University, Jeddah, Saudi Arabia; 11 Department of Epidemiology and Statistics, School of Public Health at Guangdong Medical University, Dongguan, Guangdong China; 12 Faculty of Education, Universiti Teknologi MARA, Bandar Puncak Alam, Selangor Darul Ehsan Malaysia; 13 Psychology Department, Mardin Artuklu University, Mardin, Turkey; 14 Centre for Community Health Studies (ReaCH), Faculty of Health Sciences, Universiti Kebangsaan Malaysia, Kuala Lumpur, Malaysia; 15 Non-Communicable Diseases Section, Disease Control Division, Ministry of Health, Putrajaya, Malaysia; 16 Department of Psychology, International University of Malaya-Wales, Kuala Lumpur, Malaysia; 17 Faculty of Nursing, Universitas Airlangga, Surabaya, Indonesia; 18 Filariasis Unit, Faculty of Medicine, Pharmacy and Dentistry, University of Bamako, Bamako, Mali; 19 Social Work Department, Faculty of Letters, Sivas Cumhuriyet University, Sivas, Turkey; 20 Department of Addiction, School of Behavioral Sciences and Mental Health (Tehran Institute of Psychiatry), Iran University of Medical Sciences, Tehran, Iran; 21 Social Determinants of Health Research Center, Mashhad University of Medical Sciences, Mashhad, Iran; 22 Department of Psychology, Faculty of Arts, Alexandria University, Alexandria, Egypt; 23 Department of Psychiatry, Faculty of Medicine, Universiti Kebangsaan Malaysia, Kuala Lumpur, Malaysia; 24 Centre for Healthy Ageing and Wellness (H-Care), Faculty of Health Sciences, Universiti Kebangsaan Malaysia, Kuala Lumpur, Malaysia; 25 Institute of Islam Hadhari, Universiti Kebangsaan Malaysia, Bangi, Malaysia

**Correction: BMC Public Health 22,882 (2022)**

**https://doi.org/10.1186/s12889-022-13101-3**

The original publication of this article [1] contained an error in the discussion section. The incorrect and correct information is shown below.

Incorrect:

However, a psychological autopsy study conducted on suicide deaths between July 2019 to July 2020 in Dhaka, Bangladesh showed that pesticide poisoning was still the most prevalent suicide method [47].

Correct

A psychological autopsy study conducted on suicide deaths between July 2019 to July 2020 in Dhaka, Bangladesh showed that pesticide poisoning was now the second most prevalent suicide method [47].
