# Supplementary material for: An analysis of age-standardized suicide rates in Muslim-majority countries in 2000-2019
Source: BMC Public Health. 2022 May 4;22:882. doi: 10.1186/s12889-022-13101-3 (PMC9066769; doi:10.1186/s12889-022-13101-3)
Supplement: Supplementary file 1 — Additional file 1: Fig S1. Age-standardized suicide rate in Muslim-majority countries in 2000-2019 and comparison with the global average. [file 12889_2022_13101_MOESM1_ESM.pdf]

Fig S1. Age-standardized suicide rate in Muslim-majority countries in 2000-2019 and comparison with the global average

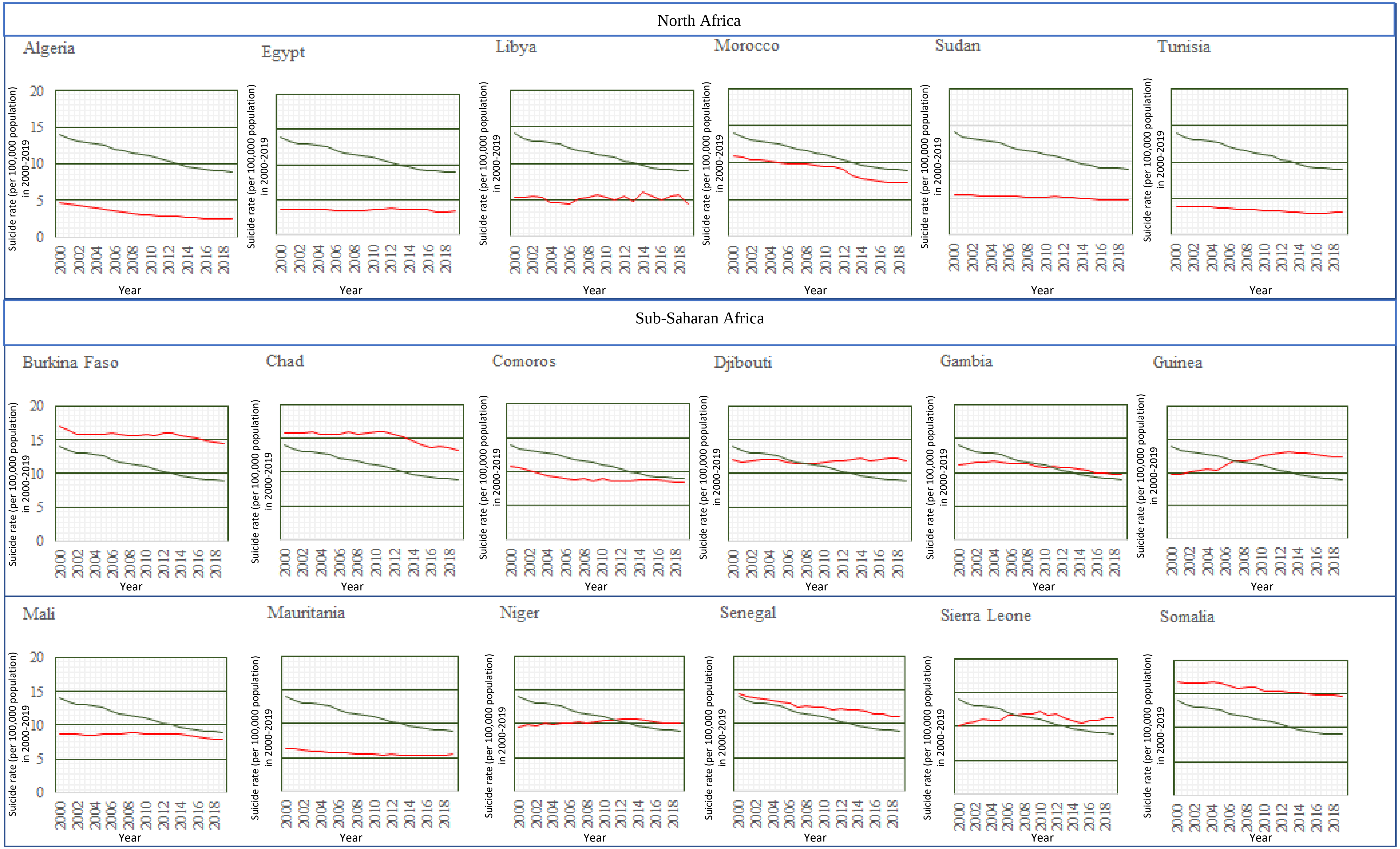

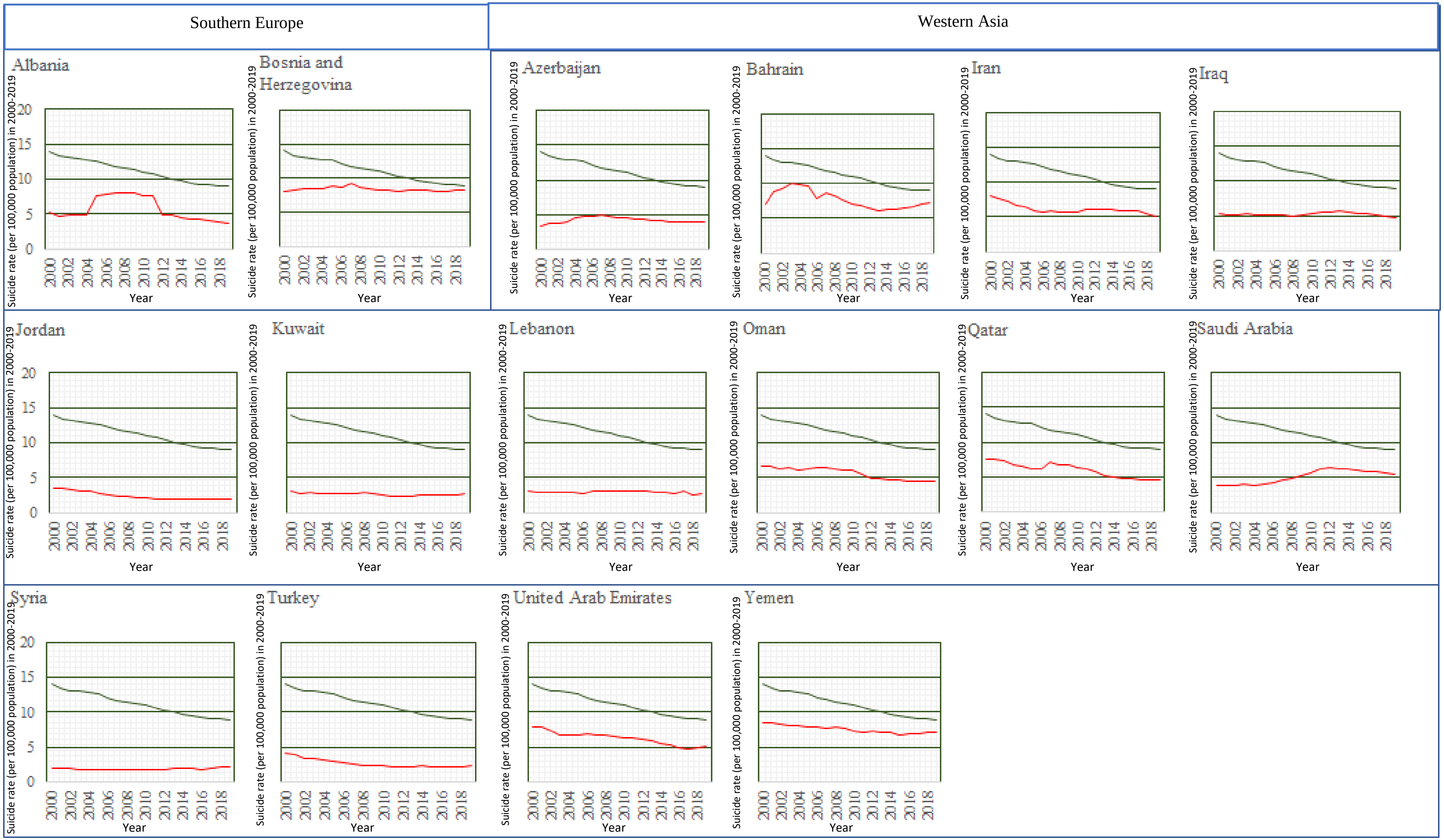
Southern Europe
Western Asia
Albania
Bosnia and Herzegovina
Azerbaijan
Bahrain
Iran
Iraq
Jordan
Kuwait
Lebanon
Oman
Qatar
Saudi Arabia
Syria
Turkey
United Arab Emirates
Yemen
Suicide rate (per 100,000 population) in 2000-2019
Year
0
5
10
15
20
2000
2002
2004
2006
2008
2010
2012
2014
2016
2018

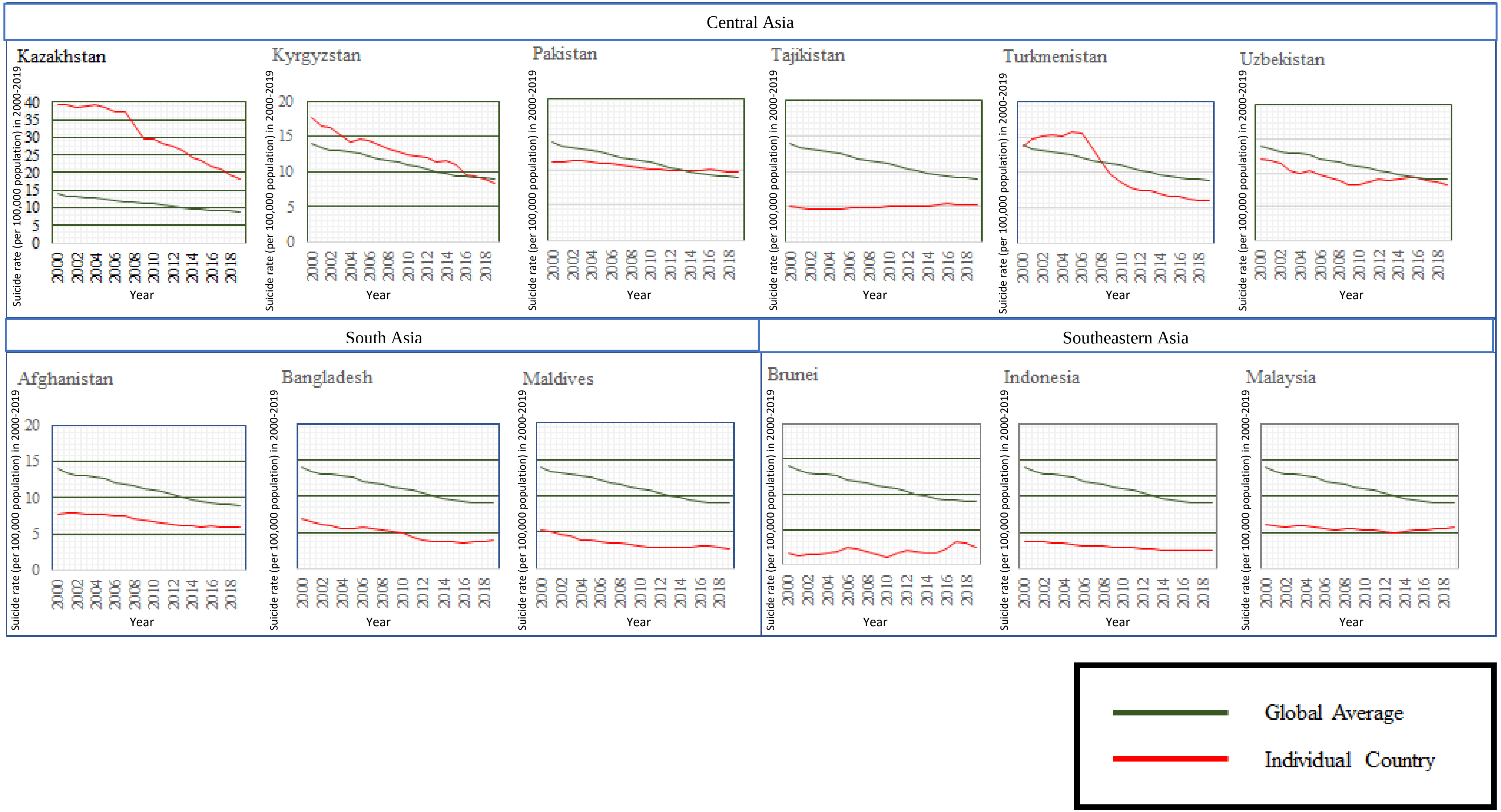

Source: WHO Global Health Estimates
